# A New Species of *Plasmodium* of the Subgenus *Novyella* Infecting White‐Shouldered Fire‐Eyes (*Pyriglena leucoptera*) (Aves: Thamnophilidae) in Brazil

**DOI:** 10.1111/1749-4877.70002

**Published:** 2025-10-09

**Authors:** Luiz Gustavo Magalhães Alves, Pedro Henrique Oliveira Pereira, Vitória Loiola Batista, Leonardo Esteves Lopes, Érika Martins Braga

**Affiliations:** ^1^ Departamento de Parasitologia, Instituto de Ciências Biológicas Universidade Federal de Minas Gerais Belo Horizonte Minas Gerais Brazil; ^2^ Laboratório de Ecologia e Ornitologia, Instituto de Ciências Biológicas e da Saúde Universidade Federal de Viçosa, Campus Florestal Florestal Minas Gerais Brazil

**Keywords:** avian haemosporidians, bird parasites, Brazil, integrative taxonomy, *Plasmodium*

## Abstract

Bird parasites belonging to the genus *Plasmodium* (*Haemosporida*: Plasmodiidae) are found worldwide, with over 50 described species categorized into five subgenera. The subgenus *Novyella* comprises 22 morphologically identified species, of which 59% are genetically associated with at least one haplotype. In the Americas, only three morphospecies have their microscopic characteristics linked to a molecular signature. In this study, we described a new species of *Plasmodium* (*Novyella*) infecting a white‐shouldered fire‐eye (*Pyriglena leucoptera*) in Brazil. Molecular analysis reveals that the new species, associated with the lineage PYLEU01, is closely genetically related to *Plasmodium* (*Novyella*) *homopolare*, exhibiting a genetic divergence of 4.18%. However, it differs from *P. homopolare* due to the presence of many mature amoeboid trophozoites and some young meronts located laterally in relation to the erythrocyte nuclei and the smaller average number of merozoites in mature erythrocytic meronts. Morphology of blood stages of new species is most similar to *Plasmodium vaughani* and *Plasmodium rouxi*, but is different from these parasites due to the presence of predominantly 4 merozoites in mature erythrocytic meronts (not characteristic of *P. vaughani*) and the presence of 5–6 merozoites in some mature erythrocytic meronts (not characteristic of *P. rouxi*). Our integrative analyses reveal that the newly described species represents a distinct *Plasmodium* parasite from other *Novyella* morphospecies.

## Graphical abstract

South America has a remarkable genetic diversity of avian haemosporidians, yet only a few species have been described combining molecular and morphological data. This study introduces *Plasmodium* (*Novyella*) *pyriglenae* sp. nov., a new species infecting the white‐shouldered fire‐eye (*Pyriglena leucoptera*) in Brazil. The sequence PYLEU01 is now linked to distinct erythrocytic morphological features.

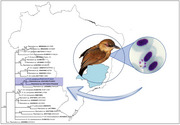

## 1 Introduction

Avian malaria parasites (Haemosporida: Plasmodiidae) are a cosmopolitan group that is widely distributed across temperate and tropical regions (Valkiūnas 2005). These heteroxenous parasites are transmitted by different species of Culicidae (Valkiūnas 2005). Free‐living birds infected with *Plasmodium* parasites may experience adverse effects on their survival and reproduction due to a trade‐off between energy and resources dedicated to immune responses and other physiological processes (Marzal et al. 2005; Arriero and Møller 2008; Martínez‐de la Puente et al. 2010). However, the pathogenicity of these parasites is often underestimated, even though infections by *Plasmodium* can be potentially fatal (Van Riper et al. 1986; Palinauskas et al. 2020), with significant conservation importance. In Hawaii, for example, the introduction of avian malaria has been responsible for severe population declines and even the extinction of many native bird species (Samuel et al. 2015)

The most well‐known genus among the haemosporidian avian parasites is *Plasmodium*, a genus that is subdivided into five subgenera*: Haemamoeba, Giovannolaia, Novyella, Huffia*, and *Bennettinia* (Valkiūnas and Iezhova 2018). The number of species included in this genus, however, is likely underestimated due to the scarcity of taxonomic studies, low taxon sampling, and cryptic speciation (Bensch et al. 2004; Palinauskas et al. 2015; Galen et al. 2018). Haemosporidian avian parasites are usually identified by examining the morphological characteristics of their erythrocytic stages in vertebrate hosts (Valkiūnas 2005; Santiago‐Alarcon et al. 2020). The phylogeny and evolutionary history of haemosporidians are usually unraveled by genetic analysis of the mitochondrial *cytb* gene (Pacheco et al. 2018).

Currently, over 50 species of *Plasmodium* that parasitize birds can be readily identified using morphological features (Valkiūnas and Iezhova 2018), but on a molecular level, over 1300 lineages are currently recognized (Bensch et al. 2009, MalAvi Database). Limited correlation between lineages and taxonomically valid species makes it difficult to accurately assess haemosporidian biodiversity (Valkiūnas et al. 2008a; Fecchio et al. 2020; Pacheco and Escalante 2023). Thus, integrative taxonomy is crucial for identifying new species correctly. The *Novyella* subgenus is the most molecularly characterized group, with 59% of its morphospecies linked to at least one genetic lineage (Valkiūnas and Iezhova 2018).

This study aims to describe a new species of *Plasmodium* of the subgenus *Novyella* discovered infecting a white‐shouldered fire‐eye (*Pyriglena leucoptera*). For that, we used morphological and molecular tools, employing Bayesian inference to assess the phylogenetic relationships of this haplotype. We also compared the newly described species with other known species of *Novyella* from South America. This work highlights the unique characteristics of the newly described species and contributes to understanding *Plasmodium* diversity.

## 2 Materials and Methods

### 2.1 Sampling Procedures

In April 2022, an adult female white‐shouldered fire‐eye *Py. leucoptera* (Aves: Thamnophilidae) was mist‐netted (Ecotone 18 × 3 m, five shelves and mesh 19 mm) in a patch of secondary semideciduous forest in the Campus Florestal of the Universidade Federal de Viçosa (19°53′18.93″S, 44°24′40.61″W, 770 m elevation), municipality of Florestal, Minas Gerais, southeastern Brazil. The white‐shouldered fire‐eye is a passerine bird endemic to the Atlantic Forest of eastern Brazil, eastern Paraguay, and extreme northeastern Argentina. This resident insectivorous bird inhabits the undergrowth of evergreen tropical forests, regularly following army‐ant swarms (Zimmer and Isler 2020).

We collected blood samples from this bird via brachial venipuncture using a sterile needle (13 × 4.5 mm). The blood sample obtained represented less than 1% of the bird's body weight and was stored on filter paper at 4°C until DNA extraction. The bird was then marked with a numbered metallic ring and released at the same place of capture. All procedures adhered to the Brazilian National Center for Bird Conservation (CEMAVE) protocols. Permits were issued by the Brazilian Biodiversity Authorization and Information System (SISBIO No. 81134‐1) and the necessary Animal Ethics Committees (CEUA‐UFMG No. 198/2022, CEUA‐UFV No. 03/2018).

### 2.2 Microscopic Examination of Blood Films

Blood samples were collected to prepare two blood smears, fixed with absolute methanol, and stained with a 10% Giemsa solution (pH 7.2) for 70 min within 72 h. An Olympus CX31 light microscope equipped with an Olympus Q‐Color5 imaging system (Olympus, Tokyo, Japan) and QCapture Pro7 imaging software (QImaging, Surrey, Canada) was used to examine blood films and capture images from samples considered to be of high quality. Two blood smears were scanned under 1000× magnification for the detection and morphological characterization of haemosporidian parasites. The intensity of parasitemia was determined by counting the number of parasitized red blood cells out of a total of 10 000 cells (Godfrey et al. 1987). Morphometric measures were performed digitally according to Valkiūnas (2005) using ImageJ software as an aid (Schneider et al. 2012). For the statistical analyses, we first assessed the normality of the morphometric measurement data and checked for homoscedasticity. For data that followed a normal distribution, we used the *t*‐test to evaluate significant differences. For data that were not normally distributed, we used the Wilcoxon test. In both cases, a *p* value of less than 0.05 was considered significant.

### 2.3 Molecular and Phylogenetic Analyses

The DNA was extracted from the blood sample stored on filter paper using the phenol‐chloroform method followed by isopropanol precipitation (Sambrook and Russell 2001). The genomic DNA pellet was resuspended in 50 µL of ultrapure water and quantified using NanoDrop 2000 (Thermo Scientific, Waltham, USA). We used a concentration of 40–80 ng/µL DNA to amplify a fragment of 478 base pairs (bp) from the cytochrome b locus (*cytb*) by a standardized nested PCR (Hellgren et al. 2004) following the sequencing and characterization of *Plasmodium* or *Haemoproteus* lineages. The primers HaemNFI (5′‐AGACATGAAATATTATGGITAAG‐3′) and HaemNR3 (5′‐GAAATAAGATAAGAAATACCATTC‐3′) were used for the first reaction, and 1 µL of this PCR product was used for the second round with the primers HaemF (5′‐CTTATGGTGTCGATATATGCATG‐3′) and HaemR2 (5′‐CGCTTATCTGGAGATTGTAATGGTT‐3′). PCR products were run on 6% polyacrylamide gels and visualized using silver nitrate staining to confirm successful amplification. The presence of bands with 478 bp indicated a positive result for parasite DNA. The PCR product was purified using polyethylene glycol 8000 (Sambrook and Russell 2001) and sequenced in both senses with dye terminator fluorescent labeling in an ABI Prism 3100 sequencer (Applied Biosystems, Foster City, USA). We verified the quality of the recovered sequence, checked for the occurrence of mixed infections, and edited the sequence using ChromasPro 2.0.6 (Technelysium Pty Ltd., Helensvale, Australia).

We conducted a Bayesian phylogenetic analysis to correlate the sequence recovered with sequences of haemosporidian species deposited in the public databases GenBank and MalAvi (Bensch et al. 2009). The sequences were aligned by MUSCLE using MEGA11 (Tamura et al. 2021), and a phylogeny was executed on Mr. Bayes 3.6. software (Ronquist et al. 2012). The best‐fit model was selected using IQ‐TREE 2.2.0 software (Minh et al. 2020), using partitioning schemes and substitution models for each nucleotide partition (TPM2u+F+I+R3; GTR+F+I+G4; TIM2+F+I+R3). We ran two Markov chains simultaneously for 5.000.000 generations, which were sampled every 100 generations. The first 12 500 trees (25%) were discarded as a burn‐in step, and the remaining trees were used to evaluate the posterior probabilities of each estimated node in the tree produced. We used the Kimura two‐parameter substitution model implemented in MEGA11 to calculate the divergence between the different lineages.

## 3 Results

Microscopic examination of the blood smears obtained from the white‐shouldered fire‐eye sampled here revealed that the bird analyzed was coinfected by *Plasmodium* (*Novyella*) sp. and *Trypanosoma* sp. (data not shown). A *Plasmodium* parasitemia of 0.12% was detected, and the smears showed all blood stages, with trophozoites and meronts being the most abundant stages. Microgametocytes and macrogametocytes were found, and although they were less abundant than trophozoites and meronts, their measurements were sufficient for morphological characterization (Table 1). After detailed morphological and molecular analysis (see below), we concluded that the *Plasmodium* (*Novyella*) found represents an undescribed species. We propose to name this new species:

**TABLE 1:** Morphometry of mature erythrocytic meronts, and gametocytes of *Plasmodium* (*Novyella*) *pyriglenae* sp. nov. (lineage PYLEU01), as well as infected/uninfected blood cells from the host white‐shouldered fire‐eye *Pyriglena leucoptera*.

Feature	Measurements (µM)
**Uninfected erythrocyte** (*n* = 31)	
Length	10.1–13.2 (11.3 ± 0.8)
Width	6.0–7.7 (6.7 ± 0.5)
Area	53.3–68.7 (62 ± 4.8)
**Uninfected erythrocyte nucleus** (*n* = 31)	
Length	5.1–6.8 (5.9 ± 0.4)
Width	2.7–3.2 (2.6 ± 0.2)
Area	14.0–18.5 (10.9 ± 1.8)
**Meront** (*n* = 23)	
Length ^§^	3.2–6.4 (4.2 ± 0.8)
Width ^§^	1.0– 4.3 (2.3 + 0.7)
Area ^§^	5.9–18.5 (10.2 ± 3.5)
Number of merozoites	2.0–6.0 (4.1 ± 0.5)
**Infected erythrocyte** (*n* = 23)	
Length	10.2–14.2 (12.1 ± 1.2)
Width	5.3–7.3 (6.2 ± 0.5)
Area	52.3–83.3 (64.2 ± 7.4)
**Infected erythrocyte nucleus** (*n* = 23)	
Length	3.3–7.2 (5.3 ± 0.9)
Width	2.2–3.4 (2.7 ± 0.3)
Area	8.1–15.2 (12.3 ± 1.8)
**Gametocyte** (*n* = 26)	
Length	10.0–14.7 (12.4 ± 1.5)
Width	1.5–3.4 (2.5 ± 0.4)
Area	23.6–44.0 (33.8 ± 5.3)
Pigment granules	4.0–12.0 (8.0 ± 2.0)
NDR ^†^	0.26–1.26 (0.58 ± 0.23)
**Gametocyte nucleus** ^‡^	Not measured
**Infected erythrocyte** (*n* = 26)	
Length	10.3–14.5 (12.3 ± 0.9)
Width	5.0–7.3 (6.4 ± 0.4)
Area	57.1–77.0 (66.1 ± 5.6)
**Infected erythrocyte nucleus** (*n* = 26)	
Length	5.1–6.4 (5.6 ± 0.3)
Width	2.1–2.7 (2.4 ± 0.1)
Area	11.2–14.3 (12.2 ± 0.8)

^†^ NDR nucleus displacement ratio according to Bennett and Campbell (1972).

^‡^ Difficult to measure feature; see text for explanation.

^§^ For meront's measurement, we only considered forms with four or more merozoites; however, we also observed forms with two and three nuclei.

*Plasmodium* (*Novyella*) *pyriglenae* sp. nov.

**Type host**: *Pyriglena leucoptera* (Vieillot, 1818) (Aves, Thamnophilidae).

**Type locality**: Campus Florestal of the Universidade Federal de Viçosa (19°53′18.93″S, 44°24′40.61″W, 770 m elevation), municipality of Florestal, Minas Gerais, southeastern Brazil.

**Type specimens**: Hapantotype (accession no. AM070 A and AM070 B) deposited in the Institute of Biological Sciences of the Universidade Federal de Minas Gerais, Belo Horizonte, Brazil. Intensity of infection is 0.12% (12 infected erythrocytes in 10 000 erythrocytes observed). Collected by L. G. M. Alves on 21 April 2022.

**DNA sequence**: Mitochondrial *cytb* lineage PYLEU01 (478 bp, GenBank accession no. PV821343).

**Site of infection**: Mature erythrocytes are the main host cells, but immature erythrocytes also might be infected occasionally.

**Prevalence**: Only one white‐shouldered fire‐eye was evaluated in this study.

**Additional hosts**: Unknown.

**Distribution**: The PYLEU01 lineage was recorded by Lacorte et al. (2013) at five study sites in Minas Gerais State and by Fecchio et al. (2021) in São Paulo State. This haplotype was exclusively detected through PCR in *Py. leucoptera*, *Dysithamnus mentalis*, and *Dysithamnus plumbeus*.

**Etymology**: The species name originates from the genus name of its type host, *Py. leucoptera*, highlighting the association between the parasite's lineage and this host.

### 3.1 Description

#### 3.1.1 Trophozoites

**FIGURE 1:**
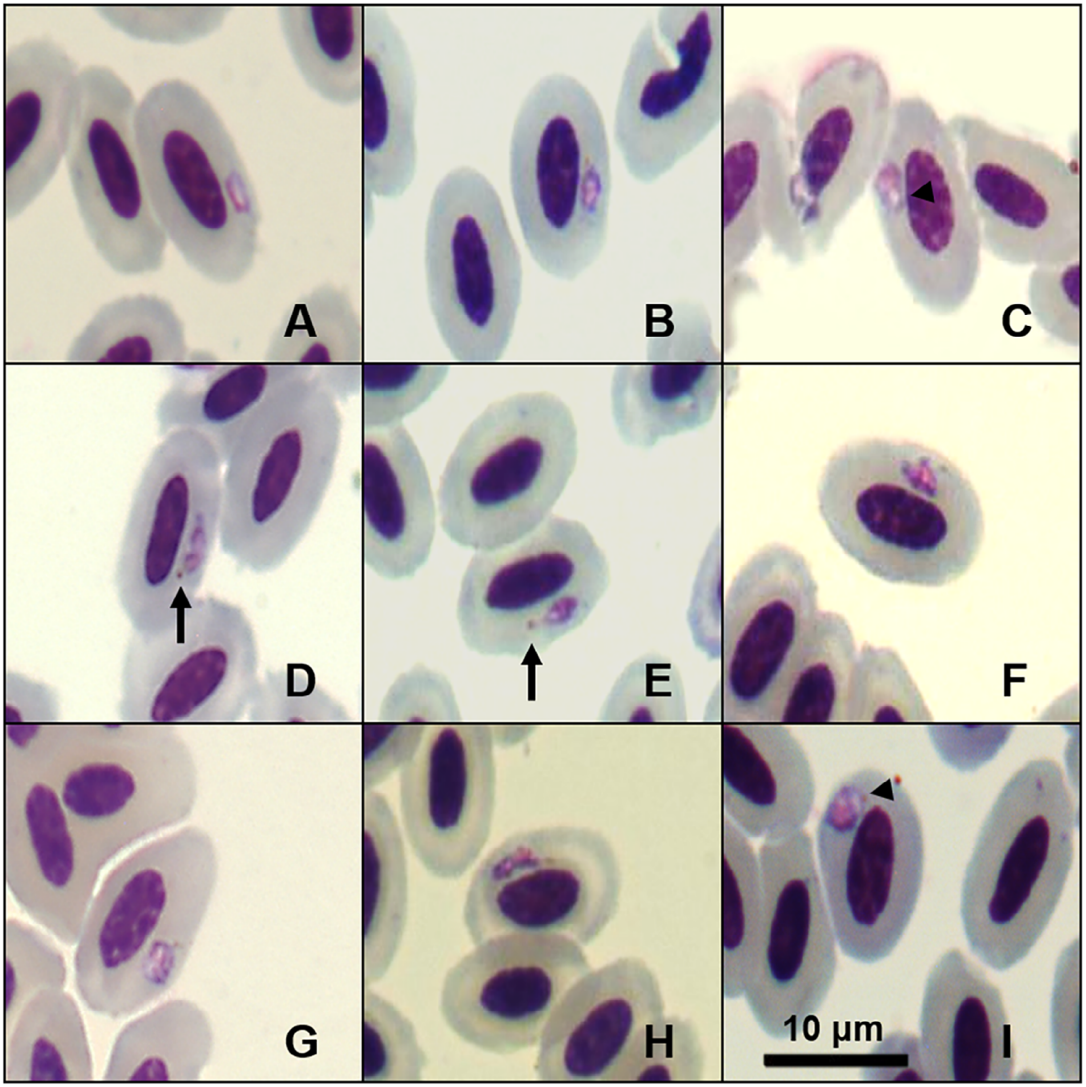
Plate exhibiting trophozoites of *Plasmodium* (*Novyella*) *pyriglenae* sp. nov. (lineage PYLEU01) from the blood smear of the white‐shouldered fire‐eye *Pyriglena leucoptera*. All images were obtained from the hapantotype specified in the remarks section. Black arrows: pigment granules; triangle arrowhead: vacuoles. Scale bar = 10 µm.

Trophozoites can be found in different locations inside the host cells, but are more often seen in a subpolar or polar position to the nucleus of mature erythrocytes (Figure 1A,C,G,I). The young forms exhibit an oval shape accompanied by a small vacuole (Figure 1C,I). We observed mature trophozoites presenting an amoeboid shape with one brown malarial pigment granule (>0.5 µm) in a polar and lateral region (Figure 1B,D,E). Trophozoites present a prominent pink nucleus, and a projection of cytoplasm may be observed (Figure 1G,H); this erythrocytic form does not contact or alter the position of the infected erythrocyte nucleus.

#### 3.1.2 Erythrocytic Meronts

**FIGURE 2:**
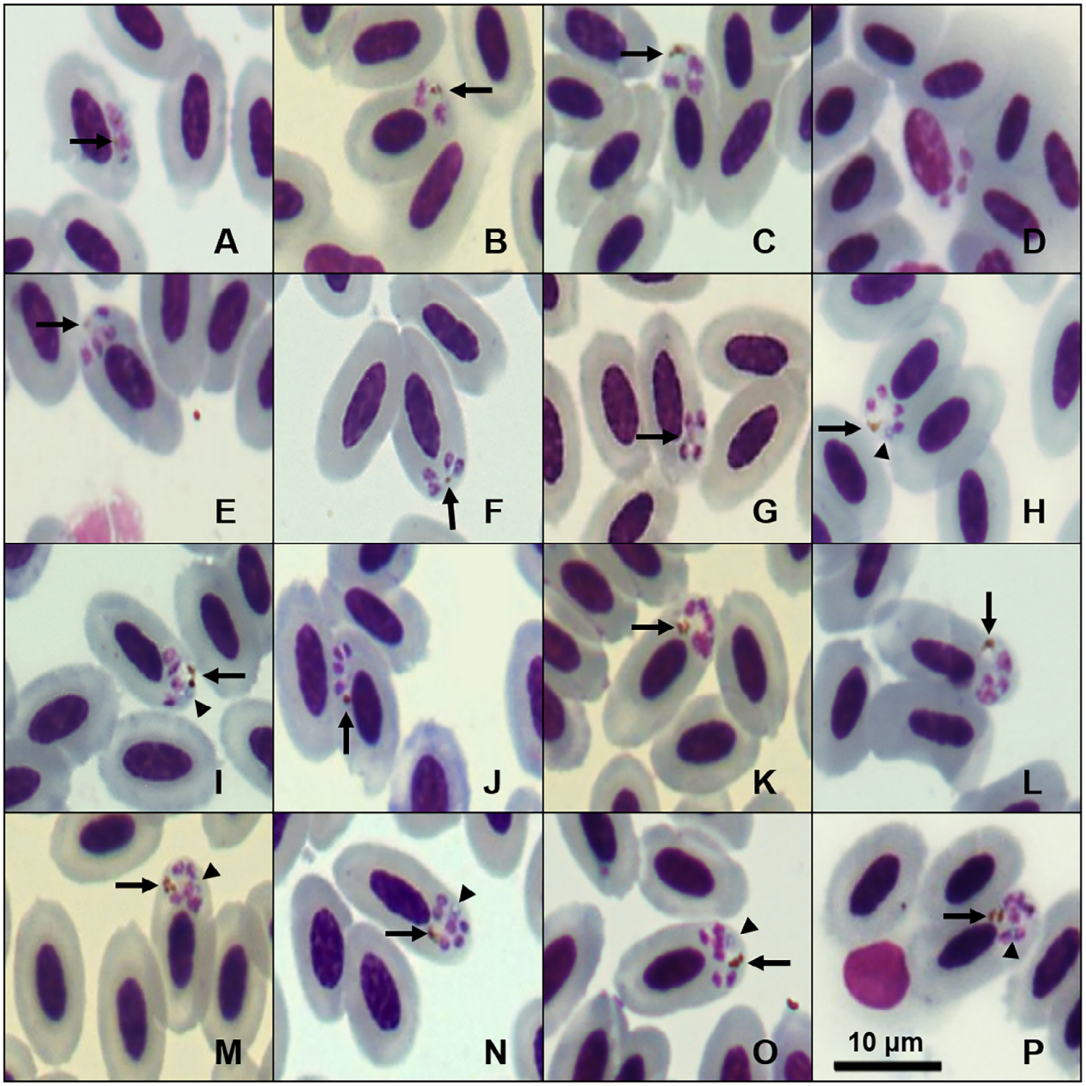
Plate exhibiting erythrocytic meronts of *Plasmodium* (*Novyella*) *pyriglenae* sp. nov. (lineage PYLEU01) from a blood smear of the white‐shouldered fire‐eye *Pyriglena leucoptera*. All images were obtained from the hapantotype specified in the remarks section. (A) Meront with two merozoites; (B,C) meront with three merozoites; (D) meront with three merozoites infecting an immature erythrocyte; (E–J) tetranucleate meronts; (K–M) meront with five merozoites; (N–P) meront with six merozoites. Black arrows: pigment granules of parasites; triangle arrowhead: bluish or colorless refractive globule. Scale bar = 10 µm.

Erythrocytic meronts, including those with a fan‐like shape, can be found in mature erythrocytes, displaying either scant or invisible cytoplasm, and they are never more prominent than the erythrocytes’ nuclei. Occasionally, we observed this parasite infecting immature erythrocytes as well (Figure 2D). Meronts are typically located in a polar or subpolar position and do not touch or displace the erythrocyte nuclei (Figure 2B–P). Infected erythrocytes are not disturbed by this stage, presenting no statistical difference from the morphological measures of uninfected erythrocytes (*p* value > 0.05). Forms that produce four merozoites (Figure 2E–J) account for 77.14% of the observed meronts. In these instances, the merozoites are typically positioned opposite to malarial pigment granules (larger than 0.5 µm; Figures 2E–H), and a blue or colorless globule (larger than 0.5 µm; Figure 2H,I,M–P), arranged in a circular pattern. Meronts with two or three merozoites (Figure 2A–D) represent 7.14% of the total, while those with five or six merozoites (Figure 2K–P) infecting mature erythrocytes account for 4.29% of the recorded forms. We observed a few meronts depicting a bow‐tie shape (Figure 2A), as described by Mohammed (1958), compared to meronts with four merozoites presenting different dispositions. We found that 72.86% of meronts contained a single malarial pigment granule, 11.43% had two, and 4.29% presented three granules. Malarial pigment granules were not identified in 11.47% of the observed meronts.

#### 3.1.3 Gametocytes

**FIGURE 3:**
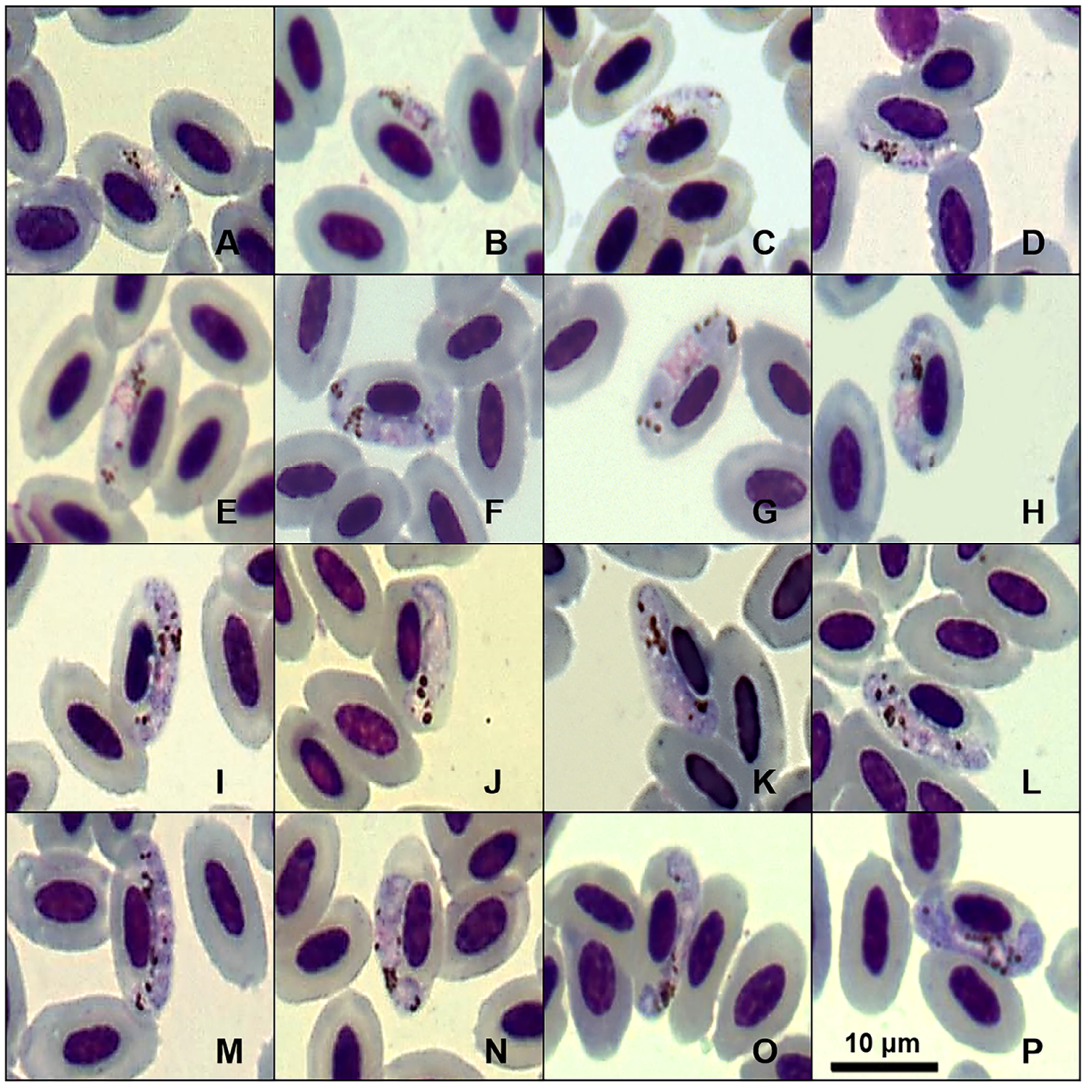
Plate exhibiting gametocytes of *Plasmodium* (*Novyella*) *pyriglenae* sp. nov. (lineage PYLEU01) from a blood smear of the white‐shouldered fire‐eye *Pyriglena leucoptera*. All images were obtained from the hapantotype specified in the remarks section. (A–E) Young gametocytes; (F–P) mature gametocytes. Scale bar = 10 µm.

Gametocytes were infecting mature erythrocytes in a lateral position, usually displacing the cell's nucleus laterally. Gametocytes have an elongated and irregular shape, with a portion touching the host cell's envelope and nucleus. Mature forms grow around the erythrocyte nucleus, filling up the host cell's poles without completely encircling them (Figure 3F–P). The gametocyte nucleus was difficult to measure due to its dispersed appearance, without a defined shape (Figure 3F,I–P). When the parasite's nucleus is visible, it is located in a central position (Figure 3E,G–H). Erythrocytes that are infected by mature gametocytes exhibit hypertrophy when compared to uninfected cells (*p* value < 0.05). Approximately 4 to 12 brownish malarial pigment granules can be found anywhere in the gametocyte cytoplasm, exhibiting an oval shape, and usually bigger than 0.5 µm.

Microgametocytes and macrogametocytes share similar features. Distinguishing microgametocytes from macrogametocytes of this new species was challenging, as they do not exhibit the classic dimorphism typically seen in haemosporidian gametocytes.

#### 3.1.4 Remarks

Based on the observed characteristics, small meronts with scanty cytoplasm and elongated mature gametocytes classify this parasite within the subgenus *Novyella*. We compared *P*. (*N*.) *pyriglenae* sp. nov. with all *Novyella* species and found that it displays distinct morphological characteristics. However, this new species typically shows most young meronts and mature trophozoites positioned laterally (Figures 1A and 2A). Although this parasite resembles *Plasmodium rouxi* (Sergent et al., 1928), the presence of meronts with five or six merozoites (Figure 2K–P) serves as a distinguishing characteristic, setting it apart from species that consistently exhibit only four merozoites. Since more than 77% of the meronts of *P*. (*N*.) *pyriglenae* sp. nov. typically produce four merozoites, a characteristic not observed in *Plasmodium vaughani* (Novy and MacNeal, 1904). This species can be clearly distinguished from others that usually have five or more merozoites, such as *Plasmodium hexamerium* (Huff, 1935) and *Plasmodium homopolare* (Walther et al., 2014). Microgametocytes and macrogametocytes are poorly distinguishable, as the observed gametocytes exhibit a pale cytoplasm and a prominent nucleus (Figure 3). Despite this characteristic, young gametocytes are frequently found in contact with the nucleus and envelope of the infected erythrocyte (Figure 3A–D), while mature gametocytes contain medium‐sized malarial pigment granules and grow toward the cell's pole, making contact with the nucleus and the envelope of the host cell (Figure 3D–P). *P*. (*N*.) *pyriglenae* sp. nov. can be differentiated from other species within *Novyella* by its frequent tetranuclear meronts, which often contain one malarial pigment granule and a bluish or colorless globule. Genetically, it shares a close relationship with *P. homopolare*, exhibiting 95.82% genetic similarity, while displaying over 5% dissimilarity from other *Novyella* morphospecies.

### 3.2 Molecular and Phylogenetic Analysis

**FIGURE 4:**
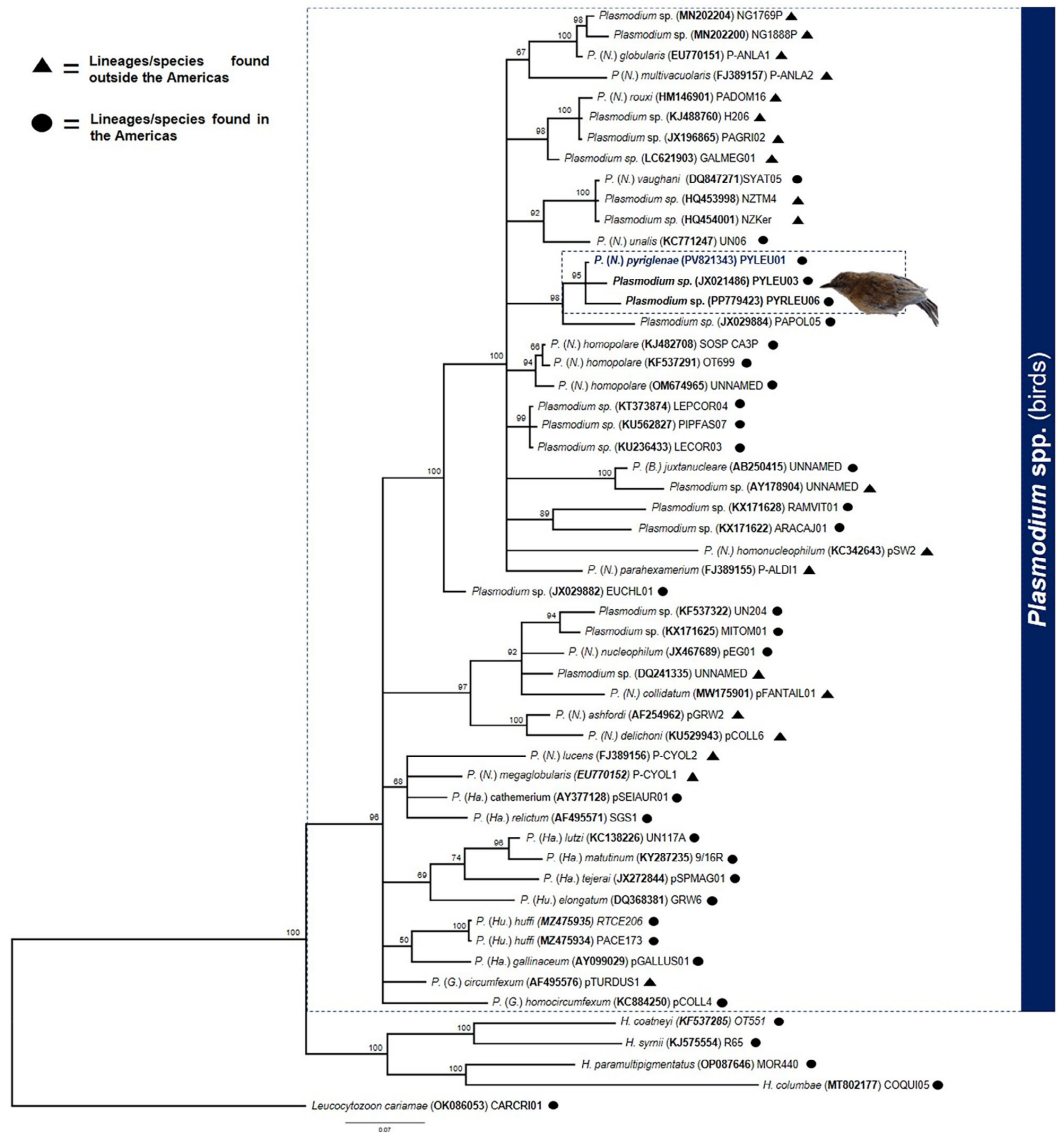
Bayesian phylogenetic inference based on 478 nucleotides of the cytochrome b gene, including *Plasmodium* (*Novyella*) *pyriglenae* sp. nov. and other *Plasmodium* spp. sequences from GenBank/MalAvi. *Leucocytozoon cariamae* CARCRI01 (accession no. OK086053) serves as the out‐group. Note that *P*. (*N*.) *pyriglenae* sp. nov. groups with *Plasmodium* lineages without morphological characterization in a distinct clade from other morphospecies of the subgenus *Novyella*.

We amplified and sequenced a 478‐bp fragment of the *cytb* gene, revealing a single *Plasmodium* sp. infection named PYLEU01 (GenBank access: PV821343). This lineage is identical to the sequence previously detected by Lacorte et al. (2013) in *Py. leucoptera* (GenBank access: JX021484). Phylogenetic analysis using partial *cytb* corroborates the results of the morphological analysis, which indicate that this parasite belongs to the *Novyella* subgenus. Our Bayesian phylogenetic analysis, which includes all avian *Plasmodium* morphospecies with their associated *cytb* sequences, as defined in the most recent key for *Plasmodium* identification (Valkiūnas and Iezhova 2018), shows that the PYLEU01 lineage is positioned within a clade mainly composed of species from the subgenus *Novyella* (see Figure S1). Additionally, we conducted an analysis that included several *Plasmodium* (*Novyella*) lineages, revealing a new clade distinct from other morphospecies (Figure 4). The pairwise distance between PYLEU01 and *Novyella* species lineages ranges from 4.5% to 10.5% (Table 2).

**TABLE 2:** Pairwise distance (in percentage) among *cytb* sequences of morphologically identified *Plasmodium* (*Novyella*) species. GenBank accession numbers are given in Figure 4.

Morphospecies	1	2	3	4	5	6	7	8	9	10	11	12	13
**1**. *P*. (*N*.) *pyriglenae* sp. nov.													
**2**. *P*. (*N*.) *homopolare*	4.5												
**3**. *P*. (*N*.) *vaughani*	5.9	4.9											
**4**. *P*. (*N*.) *unalis*	5.9	4.5	3.3										
**5**. *P*. (*N*.) *ashfordi*	10.5	7.6	8.9	8.1									
**6**. *P*. (*N*.) *delichoni*	10.2	8.9	9.7	8.4	2.8								
**7**. *P*. (*N*.) *globularis*	6.3	4.1	5.1	5.3	8.3	9.1							
**8**. *P*. (*N*.) *homonucleophilum*	7.6	7.6	7.4	6.9	9.4	9.7	8.4						
**9**. *P*. (*N*.) *lucens*	8.8	8.	8.3	8.9	9.1	9.4	8.6	9.6					
**10**. *P*. (*N*.) *megaglobularis*	9.7	9.1	9.4	8.3	8.1	8.3	9.2	9.1	6.1				
**11**. *P*. (*N*.) *multivacuolaris*	6.6	6.1	6.9	6.3	9.5	9.4	5.1	10.0	9.5	8.3			
**12**. *P*. (*N*.) *nucleophilum*	8.9	8.7	8.1	7.4	6.1	5.9	9.1	10.5	8.6	7.3	9.7		
**13**. *P*. (*N*.) *parahexamerium*	5.3	3.8	4.3	4.3	9.1	9.4	4.3	6.8	8.3	9.1	5.6	8.6	
**14**. *P*. (*N*.) *rouxi*	6.6	5.1	6.1	4.6	8.6	9.6	6.1	8.0	8.4	7.9	5.9	9.4	6.3

## 4 Discussion

South America is known for its high genetic diversity of avian haemosporidians (Clark et al. 2014). While new haplotypes are often identified in wild birds, only a few can be linked to their corresponding morphospecies. Currently, the description of new species is based on the relationship between morphological and genetic data of these parasites. This study introduced a new morphospecies, *P*. (*N*.) *pyriglenae* sp. nov., associated with the lineage PYLEU01. To date, this haplotype has been exclusively found in Brazil among Thamnophilidae, including species *Py. leucoptera*, *D. mentalis*, and *D. plumbeus* (Lacorte et al. 2013; Fecchio et al. 2021). These three species inhabit the undergrowth of evergreen tropical forests, found in the Atlantic Forest of eastern Brazil, sometimes side by side (Ridgely and Tudor 2009). Even though these two genera are included in the subfamily Thamnophilinae, they are not closely related, having diverged in the middle Miocene (Harvey et al. 2020). Thus, considering the apparent low specificity of *P*. (*N*.) *pyriglenae* sp. nov., we expect it to be found to be infecting other Thamnophilinae in the Atlantic Forest.

Despite extensive research, there is no clear standard for differentiating haemosporidian species based on their genetic identity. It is estimated that a 5% genetic divergence in a partial fragment of the *cytb* gene is sufficient to confirm that two lineages represent distinct species (Hellgren et al. 2007; Valkiūnas et al. 2009; Perkins et al. 2000). However, morphologically distinct species can exhibit 1% or less genetic divergence based on the *cytb* marker (Valkiūnas et al. 2008b). Therefore, it is crucial to exercise caution when using genetic differences between lineages as a parameter for species identification (Valkiūnas et al. 2019).

We demonstrated the competent infection of *P*. (*N*.) *pyriglenae* sp. nov. in a white‐shouldered fire‐eye through molecular studies associated with morphological description. This new parasite exhibits typical characteristics of the *Novyella* subgenus; however, mature meronts frequently contain four merozoites and exhibit a single malarial pigment granule, often accompanied by a refractile globule, which is colorless or bluish. The occurrence of meronts with five or six nuclei is less common.

Our integrative analyses reveal that the PYLEU01 shares morphological and genetic traits with a clade comprising multiple morphospecies within the subgenus *Novyella*. The newly described species is closely associated with *P. homopolare*, *Plasmodium unalis* (Mantilla et al., 2013), *P. vaughani*, and *P. nucleophilum* (Manwell, 1935). These four species exhibit distinct characteristics that reveal their taxonomic identification, particularly in relation to the morphological features of their gametocytes. *Plasmodium homopolare* has been documented exclusively in North and South America, with a specific affinity for hosts from the family Emberizidae. The 95.82% genetic similarity shared between these two lineages may reflect their distinct morphological characteristics. The typical morphology of *P. homopolare* includes meronts containing six to eight merozoites. In contrast, the newly described species shows a majority of meronts containing four merozoites, a key characteristic that helps distinguish between the two species. Furthermore, the lateral arrangement of trophozoites and meronts observed in *P*. (*N*.) *pyriglenae* sp. nov. is a notable morphological trait that diverges from the patterns associated with *P. homopolare*. These morphological and genetic divergences may indicate a differentiation reflecting varying host responses or adaptations to specific environments, which deserve more profound evolutionary studies.

The same rationale applies when comparing *P. (N.) pyriglenae* sp. nov. with *P. unalis* and *P. vaughani*. *Plasmodium unalis* was described in Colombia and is represented by the reference haplotype lineage UN227 (GenBank KC771248), with the great thrush (*Turdus fuscater*) as its type host. Similar lineages of this parasite have been reported in several countries across the Americas, including Brazil, Costa Rica, Chile, and the United States. Morphologically, *P. unalis* exhibits the typical characteristics of the subgenus *Novyella*. However, the trophozoites observed in *P. unalis* infections are round or oval, subpolar or polar, lacking cytoplasmic projections. Indeed, a genetic divergence of 5.15% between lineage UN227 and PYLEU01 further supports the evolutionary distinction between these two species. *P. vaughani* was found in South America infecting the skylark (*Alauda arvensis*); however, the type host is *Turdus migratorius*. This parasite has been recorded in bird families such as Turdidae, Sylviidae, and Petroicidae. *P. vaughani* morphospecies is associated with the cosmopolitan lineage SYAT05 (GenBank DQ847271), with occurrences also documented in Brazil. Motta et al. (2013) detected lineage SYAT05 (GenBank JF411406) in Brazil exclusively through molecular analysis of a cracid bird infected with this haplotype. *P*. (*N*.) *pyriglenae* sp. nov. can be distinguished from *P. vaughani* by the presence of a single malarial pigment granule in trophozoites. Additionally, its gametocytes extend to both poles of the erythrocyte, frequently touching the nucleus and envelope of the host cell, a characteristic not observed in *P. vaughani* or *P. unalis*. *P*. (*N*.) *pyriglenae* sp. nov. may have malarial pigment granules larger than 0.5 µm, which are often dispersed throughout the cytoplasm and sometimes clumped. In contrast, *P. unalis* typically displays clumped pigment at the poles, while *P. vaughani* features small (<0.5 µm) to medium‐sized granules (0.5 – 1.0 µm). Following the morphological characterization, the haplotype SYAT05 exhibits a genetic divergence of 5.44% compared to PYLEU01. Another example is related to *P. nucleophilum*, which has been found in Brazil, infecting a range of hosts and exhibiting morphological characteristics markedly distinct from the new morphospecies described here. This parasite displays erythrocytic stages closely appressed to the erythrocyte nucleus. Furthermore, it typically presents meronts containing six nuclei. The genetic lineage pEG01 (GenBank JX467689) shows a divergence more significant than 8%, supporting the hypothesis that lineages with more than 5% genetic divergence represent distinct morphospecies (Hellgren et al. 2007). Therefore, our phylogenetic analysis, grounded in well‐documented morphospecies and featuring a genetic divergence exceeding 5%, serves as compelling evidence for the evolutionary distinction among the lineages we studied, including UN227, SYAT05, pEG01, and PYLEU01. This significant genetic divergence highlights the uniqueness of each lineage and reinforces the importance of characterizing new haemosporidian morphospecies. It is worth mentioning that the PYLEU01 lineage clusters within a polytomic clade, indicating insufficient molecular data to resolve these phylogenetic relationships.

We incorporated additional sequences from public databases in a phylogenetic analysis to address the lack of molecular information due to reliance on morphospecies definitions. This analysis indicated that the PYLEU01 lineage forms a distinct clade, separate from the SOS‐CA3P, SYAT05, and UN227 lineages. The study also found that *P*. (*N*.) *pyriglenae* sp. nov. clusters with the PYLEU03 and PYRLEU06 lineages, all detected in the same host species, *Py. leucoptera*. Although these lineages exhibit a genetic divergence of less than 1.5%, the absence of morphological characterization creates uncertainty regarding whether they represent the same species. However, these lineages infecting the same host species may suggest a specific interaction between the parasites and Brazilian endemic birds.

In conclusion, our study applies an integrative taxonomy framework to describe, for the first time, the morphospecies associated with the PYLEU01 lineage. Phylogenetic analysis based on a partial *cytb* fragment has proven to be a valuable tool for elucidating the diversity of haemosporidian parasites (Pacheco and Escalante 2023). These findings provide compelling evidence that *P*. (*N*.) *pyriglenae* sp. nov. represents a novel species, with an evolutionary trajectory distinct from other *Novyella* species.

## Supporting information




**Figure S1** Bayesian phylogenetic inference of *Plasmodium* morphospecies based on 478 nucleotides of the cytochrome b gene. The analysis includes the newly identified *Plasmodium* (*Novyella*) *pyriglenae* sp. nov., along with all morphologically characterized *Plasmodium* species associated with sequences available in GenBank/MalAvi. *Leucocytozoon cariamae* CARCRI01 (accession no. OK086053) serves as the out‐group. *P. pyriglenae* sp. nov. groups with *Plasmodium* species primarily in the subgenus *Novyella*.
