# Supplementary figures and images for: A New Species of Plasmodium of the Subgenus Novyella Infecting White‐Shouldered Fire‐Eyes (Pyriglena leucoptera) (Aves: Thamnophilidae) in Brazil
Source: Integr Zool. 2025 Oct 9;21(1):192–202. doi: 10.1111/1749-4877.70002 (PMC12794756; doi:10.1111/1749-4877.70002)

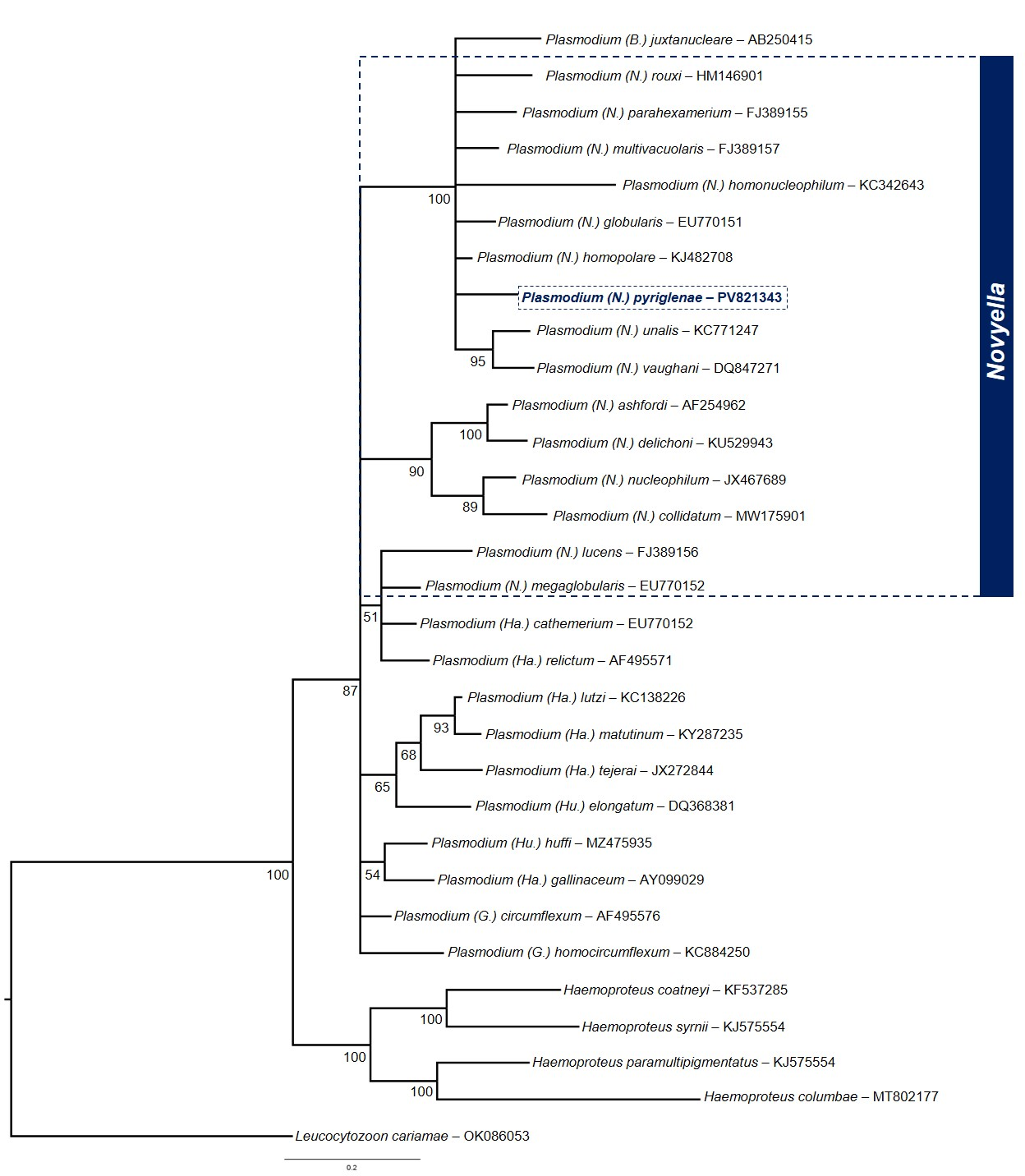

Supplement: Supplementary file 1 — Figure S1 Bayesian phylogenetic inference of Plasmodium morphospecies based on 478 nucleotides of the cytochrome b gene. The analysis includes the newly identified Plasmodium (Novyella) pyriglenae sp. nov., along with all morphologically characterized Plasmodium species associated with sequences available in GenBank/MalAvi. Leucocytozoon cariamae CARCRI01 (accession no. OK086053) serves as the out‐group. P. pyriglenae sp. nov. groups with Plasmodium species primarily in the subgenus Novyella. [file INZ2-21-192-s001.png]
